# Cytosolic 5′-Triphosphate Ended Viral Leader Transcript of Measles Virus as Activator of the RIG I-Mediated Interferon Response

**DOI:** 10.1371/journal.pone.0000279

**Published:** 2007-03-14

**Authors:** Sébastien Plumet, Florence Herschke, Jean-Marie Bourhis, Hélène Valentin, Sonia Longhi, Denis Gerlier

**Affiliations:** 1 VirPatH, Université Lyon 1, Centre National de la Recherche Scientifique (CNRS), Faculté de Médecine RTH Laennec, Lyon, France; 2 Architecture et Fonction des Macromolécules Biologiques (AFMB), Centre National de la Recherche Scientifique (CNRS), UMR 6098, Universités d'Aix-Marseille I et II, Marseille, France; 3 Immunobiologie Fondamentale et Clinique, Institut National de la Santé et de la Recherche Médicale (INSERM) U503, Université Lyon 1, IFR128 Biosciences Lyon Gerland, Lyon, France; Institut Pasteur, France

## Abstract

**Background:**

Double stranded RNA (dsRNA) is widely accepted as an RNA motif recognized as a danger signal by the cellular sentries. However, the biology of non-segmented negative strand RNA viruses, or *Mononegavirales*, is hardly compatible with the production of such dsRNA.

**Methodology and Principal Findings:**

During measles virus infection, the IFN-β gene transcription was found to be paralleled by the virus transcription, but not by the virus replication. Since the expression of every individual viral mRNA failed to activate the IFN-β gene, we postulated the involvement of the leader RNA, which is a small not capped and not polyadenylated RNA firstly transcribed by *Mononegavirales*. The measles virus leader RNA, synthesized both *in vitro* and *in vivo*, was efficient in inducing the IFN-β expression, provided that it was delivered into the cytosol as a 5′-trisphosphate ended RNA. The use of a human cell line expressing a debilitated RIG-I molecule, together with overexpression studies of wild type RIG-I, showed that the IFN-β induction by virus infection or by leader RNA required RIG-I to be functional. RIG-I binds to leader RNA independently from being 5-trisphosphate ended; while a point mutant, Q299A, predicted to establish contacts with the RNA, fails to bind to leader RNA. Since the 5′-triphosphate is required for optimal RIG-I activation but not for leader RNA binding, our data support that RIG-I is activated upon recognition of the 5′-triphosphate RNA end.

**Conclusions/Significance:**

RIG-I is proposed to recognize *Mononegavirales* transcription, which occurs in the cytosol, while scanning cytosolic RNAs, and to trigger an IFN response when encountering a free 5′-triphosphate RNA resulting from a mislocated transcription activity, which is therefore considered as the hallmark of a foreign invader.

## Introduction

The cellular innate defence is initiated with the recognition of peculiar danger molecular motifs called Pathogen Associated Molecular Patterns (PAMP), by the Pattern Recognition Receptors (PRR), which results in the induction of a type-I interferon (IFN-α/β) response. In the cytosol, the helicases RIG-I and Mda-5 induce IFN transcription upon recognition of viral dsRNA [1], [2] from different viruses, with Mda-5 and RIG-I being required for sensing picornaviruses and members of the *Mononegavirales* order [1]–[4], respectively. *Mononegavirales* are characterized by having their RNA genome (and antigenome) tightly encapsidated by the viral nucleoprotein N, making them resistant to silencing by siRNA [5], nuclease attacks or high salt concentration, as recently shown by the crystal structure of short vesicular stomatitis virus (VSV) and rabies virus nucleocapsids, which both pointed out that the RNA is fully embedded within the nucleoprotein oligomer [6], [7]. Viral genome is used as a template for transcription of unencapsidated RNAs and replication of positive stranded antigenome, the complementary template for genome replication (Figure S1). Notably, the nascent genome and antigenome are concomitantly encapsidated and hence viral complementary RNA strands are poorly prone to anneal. Indeed no detectable amounts of dsRNA have been found in infected cells [8]. We therefore searched for the PAMP and the PPR involved in the cellular detection of infection by measles virus, a *Mononegavirales member*.

## Results

### A virus transcript acts as the PAMP

**Figure 1:**
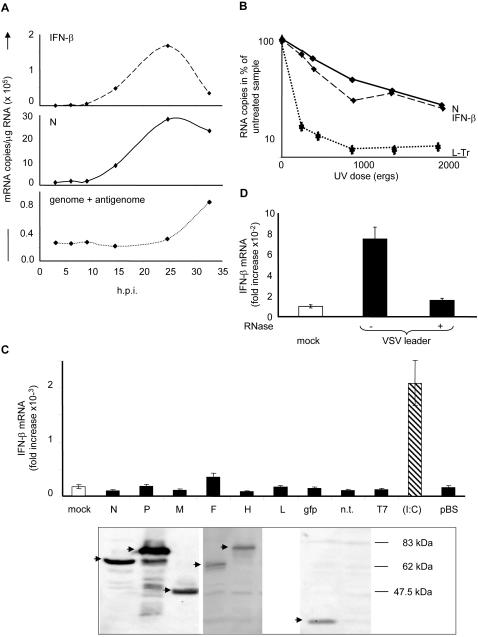
IFN-β gene activation correlates with measles virus transcription, and viral derived leader, but not viral mRNA, activates IFN-β. (A) Kinetics of the accumulation of cellular IFN-β mRNA, viral mRNA and genomes+antigenomes. 293T cells were infected with measles virus, and total RNA from cells, collected at different times post-infection, was reverse transcribed and quantified by QPCR. (B) Accumulation of cellular IFN-β and viral mRNAs after infection with virus irradiated by variable UV energies. Cells were infected for 24 h with measles virus irradiated with a range of UV energies and RNAs were quantified by RT-QPCR. Data were expressed as % of the RNA amounts from cells infected with non irradiated virus. (C) Lack of IFN-β stimulation by measles viral mRNAs. 293T cells were transfected with expression vectors (0.4 µg/10^5^ cells) encoding the various measles virus or control (eGFP, T7 RNA polymerase) proteins or with 200 ng poly(I:C). IFN-β mRNA was quantified 24 h post-transfection and protein expression was checked by western blot (see arrows). Note that, due to the lack of a suitable antibody, L protein expression could not be checked. (D) Short *in vitro* leader transcripts from VSV activates IFN-β. Identical amounts of short RNAs purified from *in vitro* transcription of VSV and treated or not with RNase A were transfected in 293T cells and IFN-β mRNA was quantified. Mock is transfection with oligofectamine alone. Results are from one representative experiment out of three.

An active viral polymerase is necessary for the induction of IFN response by measles virus (Figure 1A,B) [9]. To identify which of the two activities, transcription or replication, is required for the production of the PAMP, we compared the kinetics of accumulation of IFN-β mRNAs with the rate of measles virus transcription and replication, using RT-QPCR assays [10]. IFN-β mRNA accumulates exponentially in a manner that is paralleled by that of the viral N mRNA, well ahead of the accumulation of the genomes/antigenome (Figure 1A). The accumulation of genome/antigenome is delayed for the first 15 h (Figure 1A), until the cytoplasmic N protein concentration triggers activation of the replication [11]. Reciprocally, the progressive inactivation of viral genome by increasing doses of UV, applied to the virus before infection, results in a paralleled decrease of IFN-β and N transcription, whereas the replication process is blocked already at the lowest UV energy (Figure. 1B). When UV energy exceeds 2000 ergs, both viral and IFN-β transcriptions are abolished [9], [11].

However, none of the individual capped and polyadenylated viral mRNA *per se* contains any molecular motif recognized as a PAMP, since their transient expression in 293T cells do not lead to the induction of any IFN-β signal (Figure. 1C). This also rules out that any viral protein is responsible for the IFN-β activation, in agreement with a previous report showing that protein synthesis is not required [12].

### Viral leader RNA activates the IFN-β response

We hypothesized that the leader RNA [13] may host a PAMP feature. Leader is the first transcript from the very 3′end of the genome, and is 56 nucleotides long, not capped, and not translated. This first transcript could fulfil the requirements of the rapid induction of the IFN system after infection, as shown by the quick phosphorylation of I-κB, one of the upstream signals of the IFN-β gene activation, which is observed as early as 5 minutes after virus entry [14], and by the NF-κB DNA binding activity detected within one hour [12].

**Figure 2:**
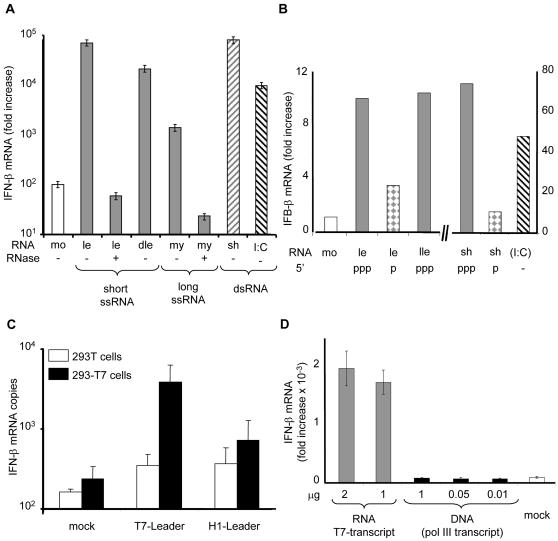
5′-triphosphate ended RNA are strong activators of IFN-β. (A) *In vitro* T7 transcripts activate the IFN response independently of RNA sequence 293T cells were transfected for 24 h with 200 ng of T7 transcripts purified according to their size: short single strand 56 nt leader (le), 56 nt delear (dle, an anagram from leader with poor predicted structure, see Figure S3), single strand 86 nt myc (my) RNA, 62 nt shRNA^eGFP^ (sh) or poly(I:C); (mo) = mock. As controls, RNAs were digested with RNase A before transfection. (B) 5′-triphosphate RNA are stronger IFN inducers than 5′-monophosphate RNA. Monophosphate RNAs were obtained as described in Figure S3 and have length identical to their 5′triphosphate counterparts. 200 ng of 5′-triphosphate short leader (le), 5′-monophosphate short leader (le), 5′-triphosphate long leader (lle), 5′-triphosphate-shRNA^eGFP^, 5′-monophosphate-shRNA^eGFP^ (sh) or poly(I:C), were transfected into 293T cells. The results are from one representative experiment out of three. (C) *In vivo* T7-driven, but not *in vivo* Pol III-driven transcription of measles virus RNA leader activates IFN-β. 293T and 293-3-46 (noted 293-T7) cell line stably expressing the T7 polymerase were transfected with 0.4 µg/10^5^ cells of pcDNA-t7-leader or pSUPER-H1-leader and the amount of IFN-β transcripts was quantified. (D) *In vitro* T7 transcripts but not *in vivo* Pol III transcripts induce IFN-β activation. 293T cells were transfected with peGFPN2 plasmid coding for the eGFP together with either eGFP specific shRNA transcripts made *in vitro* by the T7 polymerase or a plasmid DNA encoding an eGFP specific shRNA under the Pol III promoter.

The *in vitro* transcription of measles virus is hardly efficient [13]. Therefore, we first chose to produce *in vitro* the leader RNA (47 nt long) from permeabilized VSV virions, since it is biochemically indistinguishable from that isolated from VSV infected cells [15], [16]. Upon transfection, a VSV leader preparation stimulates IFN-β gene transcription, while the IFN response is abolished when the sample was pretreated with RNase A (Figure 1D). Thus, a *Mononegavirales* leader RNA, synthesized by the viral polymerase, can act as a PAMP. To demonstrate that the measles virus leader can induce an IFN response, a leader-like RNA was transcribed *in vitro* by the T7 phage DNA-dependent-RNA-polymerase from an engineered plasmid. This single strand leader RNA turned out to be a strong inducer of the IFN-β transcription in 293T cells (Figure 2A, lE) with the PAMP activity being destroyed after RNase treatment.

### 5′-triphosphate end is required for optimal activation of IFN-β

Measles virus and VSV leaders share a common 5′-triphosphate structure that could account for IFN activation. Indeed, the role of the 5′-triphosphate end of T7 transcripts as an IFN inducer had been previously pointed out for shRNA [17]. Using different T7 transcripts, including a “delear” 56 nt long RNA (dle)-which has the same bases as the leader, but in a scrambled order-a myc tag sequence 86 nt long RNA (my) and a small hairpin 62 nt long RNA with 21 base-pairing of the coding sequences of eGFP (shRNA^eGFP^), we confirmed that T7 transcripts, independently of their length, sequence or structure (Figure S2), do possess PAMPs (Figure 2A). Furthermore, converting the triphosphate into a monophosphate moiety at the 5′end of leader and shRNA^eGFP^ RNAs before transfection, either by calf alkaline phosphatase treatment (data not shown) or by RNase H digestion of a longer RNA precursor partially annealed at the 5′end to a short complementary DNA (Figure S3), reduces their ability to activate the IFN response (Figure 2B). Thus, 5′-triphosphate represents a PAMP even on short single strand RNA (ssRNA). To mimic viral synthesis of measles viral leader in the cytoplasm, cells stably expressing cytosolic T7 phage polymerase [18] were transfected with a plasmid encoding the viral leader under the control of the T7 promoter. As a control, the leader was alternatively put under the control of the H1 promoter, which is recognised by the cellular RNA-polymerase III. IFN-β was significantly induced only when the viral leader was transcribed by the cytosolic T7, and not by the nuclear endogenous RNA polymerase III (Figure 2C). Likewise, shRNA^eGFP^ transcribed in the nucleus by the cellular RNA-polymerase III after transfection of pSUPER-shGFP DNA plasmid fails to activate the IFN-β response. Expression of shRNA^eGFP^ was assessed by its ability to silence eGFP expression [19]: transfection of 0.01 µg of pSUPER-shGFP DNA plasmid was found to be as efficient as transfection of 2 µg of shRNA^eGFP^ to silence the eGFP expression. While up to 2 µg of the former does not activate any IFN-β response, transfection of 1 or 2 µg of the latter strongly activates IFN-β (Figure 2A,D).

What would be the *in vivo* relevance of the recognition of the 5′-triphosphate motif in the cytosol as a danger signal? A major feature of the nuclear (and mitochondrial) transcription, by either RNA polymerase I, II or III (and mitochondrial RNA-polymerase), is that all, but one, RNAs are processed at the 5′end either by cleavage (ribosomal RNA, tRNAs, microRNAs) or by a capping process resulting into the loss of the primary 5′-triphosphate end [20]–[22]. The only known exception is the abundant 7SL RNA that is part of the signal recognition particle, but its 5′end is likely inaccessible as it is shielded by the p9/14 protein subunit [23]. Accordingly, cytoplasmic and nuclear RNA extracted from non infected 293T cells were found to be incapable of inducing any IFN-β response after transfection (Figure S4). Furthermore, cellular RNA transcription from RNA template does not occur in mammalian cells because of the lack of RNA-dependent RNA-polymerase cellular homologue. A 5′-triphosphate ended RNA in the cytoplasm thus represents a well-suited feature for a danger signal, i.e. a molecular pattern that is absent from uninfected cells according to the concept described by P. Matzinger [24], and we propose that it acts as a PAMP used by cells to recognise a virus infection at the very early steps.

### RIG-I is required for recognition of measles virus infection and sensing 5′-triphosphate ended RNA transcript

**Figure 3:**
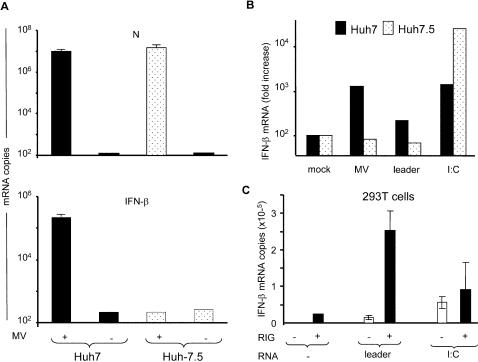
IFN-β activation by measles virus and by viral 5′-triphosphate leader is mediated by RIG-I. Lack of IFN-β response in the RIG-I deficient Huh7.5 cells after measles virus infection (A) or transfection with 200 ng of *in vitro* T7-driven leader transcript (B). Huh7 and Huh7.5 cells were infected with measles virus for 24 h and checked for N and IFN-β mRNA expression. (C) Exogenous expression of Flag-RIG-I enhances IFN-β stimulation by *in vitro* T7-driven leader transcript. 293T cells were transfected with a control plasmid or a plasmid coding RIG-I (0.4 µg DNA), followed 24 h later by transfection with no RNA, T7 transcribed leader or poly(I:C) (200 ng). 24 h after the second transfection, IFN-β mRNA was quantified as described above. Ectopic expression of Flag-RIG-I was controlled by western blotting (not shown).

Since RIG-I has been associated to the sensing of infection by other *Mononegavirales* [1]–[4], we tested if RIG-I could also sense measles virus 5′-triphosphate leader RNA. To ensure a homogenous defect in RIG-I of the cell host, we took advantage of the Huh7.5 cell sub-line derived from Huh7 cells. Huh7.5 expresses a debilitated RIG-I with a point mutation in the first CARD domain (T55I), which no longer produces IFN after infection by VSV or SeV unless complemented by exogenous wild type RIG-I [25]. Measles virus infection triggers the IFN response in human Huh7 cells, but not in the RIG-I defective Huh7.5 cells, although the accumulation of N mRNA reached a similar level in both cell lines after 24 h (Figure 3A lower and upper panel, respectively). Accordingly, Huh7.5 cells fail to respond the *in vitro* T7 transcribed leader RNA (Figure 3B). However, IFN-β is activated in Huh7.5 by the dsRNA analogue poly(I:C), showing than the Mda-5-mediated and the IPS-1 (also called MAVS, CARDIF or VISA) downstream pathway, which binds to both RIG-I and Mda5 to ensure a common signalling [26], [27], is functional in this cell line. The inefficiency of the Mda-5 pathway of Huh7.5 cells to respond to measles virus infection is likely because of the Mda-5 inhibitory properties of the *Paramyxoviridae* (including measles virus) V protein [28], [29]. Moreover, the co-transfection of RIG-I with leader RNA has a strong synergic effect on the induction of IFN-β transcription, the combination resulting in more than 10-fold higher IFN-β accumulation than transfection of RIG-I or leader RNA alone (Figure 3C). In contrast, the over-expression of RIG-I had little or no enhancing effect on the IFN-β induction by poly-(I:C) [4].

### RIG-I binds to leader RNA independently of RIG-I ATPase activity and of RNA tri-or mono-phosphate 5′end

**Figure 4:**
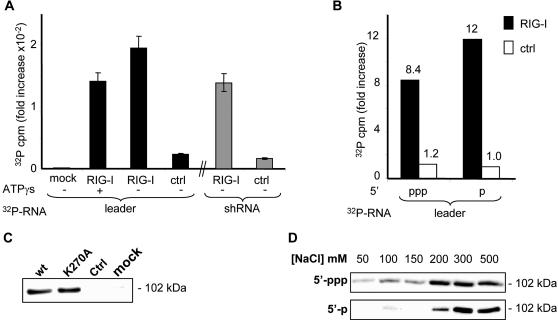
**Binding of** *in vitro* transcribed leader RNAs to RIG-I. (A) Anti-Flag M2 immunoprecipitates from cytoplasmic extracts of cells over-expressing Flag RIG-I or Redfp proteins as control (ctrl) were incubated with [^32^P]-labelled RNA leader or shRNA transcripts made *in vitro* by the T7 polymerase, in the presence or absence of non hydrolysable ATPγs. Western blotting using Anti-Flag M2 antibody were used to assess the precipitation of Flag-RIG-I on the beads (not shown). (B) A similar experiment was performed with radio-labelled 5′-triphosphate or 5′-monophosphate leader transcribed *in vitro* by T7 polymerase. Mock (mo): beads incubated with [^32^P]-radio-labelled RNA in the absence of cytoplasmic extracts. (C) Extracts of cells over-expressing FlagRIG-I (noted wt), FlagRIG-I[K270A] or Redfp as control (ctrl) were incubated with biotinylated 5′-triphosphate measles virus leader RNA, and the complex was then pulled-down with Streptavidin-coupled beads. Proteins were eluted with 500 mM NaCl and analysed by Western blot for RIG-I binding to RNA. Mock represents incubation of RNA without protein. (D) Step gradient elution of FlagRIG-I with either 5′-triphosphate (5′-ppp) or 5′-monophosphate (5′-p) biotinylated measles virus leader RNA. Proteins were sequentially eluted with increasing NaCl concentrations, and analysed for bound RIG-I by western blot.

The ability of RIG-I to bind to the leader RNA was then tested *in vitro*. Ten times more [^32^P]-labelled leader were pulled down by RIG-I when compared to a mock immunoprecipitate (Figure 4A). Moreover, binding is independent from the ATPase activity of the helicase. RIG-I pull-down of [^32^P]-labelled T7-driven transcripts is indeed poorly affected by the presence of the non hydrolysable ATPγs, and an ATPase-defective RIG-I mutant, K270A, efficiently binds to [^32^P]-labelled T7-driven transcripts (data not shown) and to biotinylated leader RNA (Figure 4A and C). [^32^P]-labelled microRNAs, purified from *in vitro* VSV transcription, are also specifically pulled down by RIG-I (data not shown). However, the mono-or tri-phosphate status of the 5′ end of RNA does not strongly influence the RNA binding ability of RIG-I (Figure 4B), in agreement with the reported ability of dsRNA to pull down RIG-I [1]. Moreover, the RIG-I affinity for 5′-triphosphate and 5′-monophosphate leader RNA is similar, as grossly judged on the basis of saline elution step gradient (Figure 4D). Thus, the 5′-triphosphate appears to be dispensable for RNA binding to RIG-I, but it is involved in RIG-I activation.

### Modelling of the RIG-I helicase domain and prediction of Q299 as an RNA contacting residue

**Figure 5:**
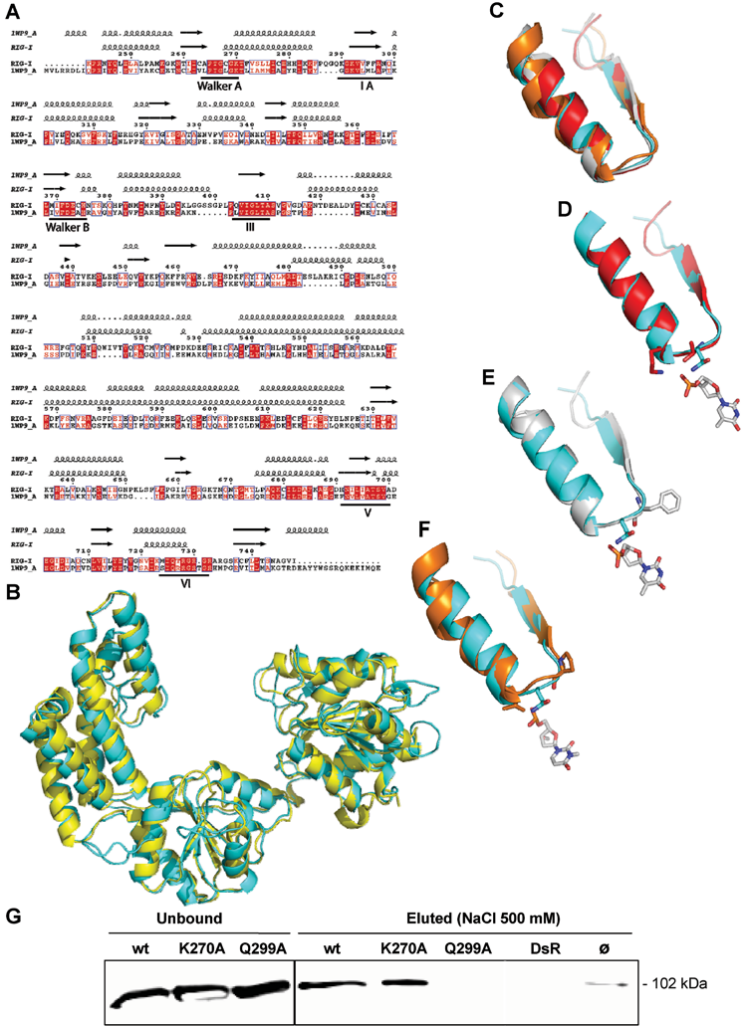
Structural model of the helicase domain of RIG-I, prediction of Gln299 as an RNA binding residue and validation by RNA binding assays. (A) Sequence alignment of the RIG-I helicase domain (residues 242–763) and of Hef (1WP9, residues 1–494). Protein sequences were aligned using the program MUSCLE [51], and manually refined. Predicted and actual secondary structure elements of RIG-I and Hef are shown above the alignment. Sequence numbering corresponds to the RIG-I sequence. The dots in the alignment and in the structural elements indicate gaps. Identical amino acids are boxed in red. Similar residues are drawn in red. The seven helicase motifs are underlined. (B) Superimposition between the RIG-I homology-derived 3D model (cyan) and the crystal structure of the *Pyrococcus furiosus* helicase (yellow). (C) Superimposition of the motif Ia from Rep (red), PcrA (white), HCV NS3 (orange) and RIG-I (cyan). (D, E, F) Superimposition of RIG-I motif Ia onto that of Rep (D), PcrA (E), and HCV NS3 (F). The residues contacting the nucleic acid are shown in sticks. The base closest to the Ia motif in each helicase is shown in white and in sticks. (D) Stick representation of Rep Thr56 and Lys 58 (red), and of RIG-I Gln 299 (cyan). (E) Stick representation of PcrA Phe64 (white) and of RIG-I Gln299 (cyan). (F) Stick representation of NS3 HCV Pro230 and Val232 (orange), and of RIG-I Gln299 (cyan). (G) Loss of RIG-I binding to RNA after Gln299Ala mutation. Binding assays were carried out as described in Fig. 4 legend.

In order to obtain mechanistic insights into the RIG-I interaction with RNA, we searched the pdb for possible structural homologues. This led to the identification of Hef, a helicase from *Pirococcus furiosus* [30] that shares 43% similarity and 25% identity with the helicase domain of RIG-I. After refining the sequence alignment between RIG-I and Hef (Figure 5A), we used it as a template to build a homology-derived 3D model of the helicase domain of RIG-I (Figure 5B).

In order to identify the RIG-I residues possibly involved in RNA binding, we performed a structural alignment among the RIG-I model, and the crystal structures of PcrA (a DNA helicase from *Bacillus stearothermophilus*) [31], of Rep (a DNA helicase from *E. coli*) [32] and of the hepatitis C virus (HCV) NS3 helicase [33]. Notably, PcrA and Rep, which share an identity of 42%, have been crystallised with a DNA oligonucleotide [31], [32], while the crystal structure of the HCV NS3 contains an RNA molecule [33]. Although NS3 has a more divergent primary sequence, it has an overall fold very similar to that of Rep and PcrA [34].

Among the nucleic acid binding motifs of helicases (namely motifs Ia, III, IV, V), the motif Ia is devoted to oligonucleotide binding, while the other motifs have also additional and not yet well defined functions [35]. As these motifs are divergent in their sequence, the oligonucleotide-binding residues are not conserved [35]. Moreover, even when the motifs are strictly conserved, as is the case of motif Ia (FTNKAA) in PcrA [31] and Rep [32], the residues involved in DNA binding are not the same (Phe64 in PcrA, and Thr56 plus Lys58 in Rep). In addition, despite the overall very similar fold shared by these helicases, the bound oligonucleotide molecules are not superimposable (data not shown). Altogether, these latter points highlight the divergence of helicases in their mode of binding to the nucleic acid.

Despite the low overall sequence identity (less than 15%), a very good superimposition of the secondary structure elements of the Ia motif was found among the four structures (with a rmsd of 0.5–1.0 Å) (Figure 5C). In order to identify the RIG-I residue(s) possibly involved in the interaction with RNA, we analysed the residue(s) involved in the interaction with the oligonucleotide in the three helicases, and their structural counterpart in RIG-I.

In Rep, two residues interact with the DNA, namely Thr56 via its hydroxyl group, and Lys58 via its amino group (Figure 5D). In the RIG-I model, the only residues close (<4 Å) to the DNA molecule crystallized with Rep are Gln299 and Pro301. These latter residues are also close enough to establish contacts with the DNA molecule of the PcrA-DNA complex, although the PcrA DNA binding residue is a phenylalanine (Phe64) (Figure 5E). Within HCV NS3, two residues (namely Pro230 and Val232) interact with the RNA molecule (Figure 5F). Within RIG-I, there are three residues that could interact with the RNA molecule: Tyr303, Ile300 and Gln299. Tyr303 could establish an H bond via its OH group to the alpha phosphate of this RNA, and Ile300 could interact either by stacking the ribose (or base) cycle, or through its backbone carbonyl or amide atoms. The side-chain amide group of Gln can act simultaneously as hydrogen bond donor and acceptor. Gln299 is the polar residue closest to RNA and could more specifically interact (i.e. via its side chain) with the nucleic acid.

In conclusion, the structural comparison led us to predict Gln299 as the best located residue to specifically interact with RNA. Accordingly, we designed and constructed a RIG-I Q299A mutant, and found that the mutated protein is unable to bind significantly to biotinylated leader RNA (Figure 5G).

## Discussion

Our present findings strongly support 5′-triphosphate ended ssRNAs as being a danger signal capable of inducing cellular innate immunity. These ssRNAs are recognised by the helicase RIG-I.

### Mechanism of RIG-I binding to RNA and activation

The bioinformatics analysis of the RIG-I sequence, as well as RNA binding experiments, confirm previous findings [1] indicating that this molecule belongs to the DExD/H RNA helicase family. RNA binding is mediated by the helicase domain [1], [36] and we showed that it involves the Gln299 within the helicase Ia motif, while the ATPase site does not play a crucial role. RIG-I has been reported to bind to many different ssRNA and dsRNA ([1], [36], [37] and this work). Using a physiological RNA target for RIG-I, the measles virus leader RNA, *in vitro* RNA binding to RIG-I was found not to be significantly enhanced by the presence of a 5′-triphosphate end in contrast with the recent reports from Hornung *et al.* and Pilchmair *et al.* who carried out similar assays [36], [37]. A possible reason for this discrepancy could be that the longer ssRNA (56 nt) we used, as compared to those used by Hornung et al. (24 and 31 nt) [36] could obscure the contribution of the 5′ triphosphate end to the RNA binding. Pilchmair *et al.* [37] used much longer RNAs (356 nt) and a RIG-I-GFP fusion protein instead of Flag-tagged RIG-I protein. We could speculate that the presence of the GFP tag may lead to subtle conformational changes of the helicase domain, possibly by modifying its post-translational modification through changes in the cellular polyubiquitination system [38]. Since alanine substitution of Gln299, predicted to bind to the RNA chain and not to the 5′-triphosphate end, abolishes the ability of RIG-I to bind to RNA, we propose that the 5′ end of RNA only marginally contributes to RNA binding, while it is critical for RIG-I activation and exposure of CARD domains for binding to IPS-1. Indeed, isolated CARD domains from RIG-I act as constitutive activator of the IFN response [1].

### Short uncapped measles virus RNA leader transcript as activator of the RIG-I-mediated IFN response

As other *Mononegavirales*, measles virus has a polymerase producing two types of 5′-triphosphate RNAs [39], [40], the 15,894 nt long genomic and antigenomic RNAs, and the short leader RNA which could be recognised by RIG-I. The 5′-triphosphate moieties of genome and antigenome are not recognized, as shown by the inability of UV inactivated measles virus to induce an interferon response ([9] and our data), likely because they are shielded within the nucleocapsid [41]. Moreover, during virus replication, the simultaneous encapsidation by the N protein of nascent 5′-triphosphate genome/antigenome ends is predicted to prevent them from being recognised by RIG-I. As a consequence, if, as recently described [36], the 5′-triphosphate moieties of naked genomes and antigenomes from rabies virus, a *Mononegavirales*, can be recognized by RIG-I, this is unlikely to reflect the *in vivo* situation. Rather, we propose that the short 5′-triphosphate leader RNAs transcribed in the cytosol by all *Mononegavirales* members but *Bornaviridae*, which replicate into the nucleus, represents a danger signal that triggers the RIG-I mediated innate immunity and accounts for the massive and immediate IFN induction after infection. Although 5′-triphosphate ended leader RNA from measles virus has the potential of being responsible for the activation of the RIG-I dependent IFN-β activation, it remains to be established whether this small RNA is produced as free RNA in significant amounts during virus transcription. So far, measles virus leader RNAs have been found only as encapsidated read-through leader-N gene RNAs, and they should be poorly prone to activate RIG-I, based on the same arguments as for the genomes/antigenomes. From the current model [42] mostly derived from studies done on Sendai virus and VSV, replication and transcription promoters of *Mononegavirales* are always switched on and their polymerases continuously enter at the 3′end of genomes and antigenomes to initiate RNA synthesis. In the absence of soluble N protein, the polymerase is poorly processive and produces small amounts of leader and a larger amount of leader-N readthrough short RNAs [43] which fall off the template because of the poor processivity of the polymerase. Upon scanning forward and backward, the polymerase encounters the transcription promoter located downstream of the genomic promoter and starts the transcription of the first N gene. Transcription becomes processive because the nascent mRNA is capped by the polymerase shortly after the initiation of the mRNA synthesis. Upon arriving at the next intergenic junction, the polymerase stutters to add polyA, stops and restarts to synthesize the next gene. Later in the infection, the availability of soluble N allows the specific encapsidation of nascent leader sequence which contains the encapsidation signal, and switches the polymerase to a processive replicase mode. Indeed, most of the read-through leader-N RNAs are also found encapsidated at later times after infection [43]–[45]. Although a similar RNA synthesis initiation can occur on the antigenome with the synthesis of a short trailer transcript, it requires the accumulation of viral antigenomes [46] that begins 12–15 h post-infection [11]. According to this model, 5′-triphosphate ended leader sequences are continuously transcribed at a level similar to the transcription of the first N gene and as such remain the best candidate for the RIG-I-dependent stimulation of the IFN-β response. The lack of detection of such small non encapsidated transcripts in the case of measles virus [45] may reflect their high instability and/or their rapid dismantling after their interaction with RIG-I.

Finally, from a co-evolution point of view, our and other data [1]–[7], [36], [37] suggest that mammalian cells have evolved so as to confine transcription and various RNA maturation processes within cellular organelles. As a result, this has enabled cells to develop new tools to detect any RNA synthesis occurring in the cytosol such as a pathogen transcriptional activity. Such RNAs, being 5′-triphosphate ended, are readily recognised by RIG-I. As a result, RNA viruses completing transcription in the cytosol have developed various strategies such as efficient capping process either by cap-snatching (*Orthomyxoviridae*) or capping activities of their polymerases (*Mononegavirales*), shielding nascent genome and antigenome in the nucleocapsid (*Mononegavirales*) or efficient coupling of nascent 5′end RNA with a protein which is necessary to prime the viral polymerase (*Picornaviridae*). In addition they have developed many other strategies to counteract the IFN activation pathway (see [47] for review).

## Methods

### Cell lines and viruses

Human kidney 293T/17 and liver Huh7 and Huh7.5 epithelial cell lines, measles virus Hallé and VSV Indiana strains were used and maintained as reported [11]. In all experiments, measles virus infections were performed at a multiplicity of infection of 1. In the case of UV irradiation, this infectivity was determined before this treatment.

### Plasmids

Plasmids used were pSC6-N, pSC6-P, pSC-M, pcX2N-F, pcX2N-EdH, pRSV-L, pegfpN2 (Invitrogen), pSC6-T7, pDsRed (Invitrogen), pEF-BOS-RIG-I [1] coding for measles virus-N, -P, -F, -H, -L, eGFP, T7 polymerase, Redfp, Flag-RIG-I, respectively.

### *In vitr*o transcription and RNA purification

*In vitro* transcriptions were performed on 1 µg of linearized plasmids to get the exact length of RNA transcripts using Ribomax large scale *in vitro* Transcription System-T7 (Promega). pSUPER-leader, pegfpN2-leader, pegfpN2-delear, pegfpN2-trailer, and pegfpN2-longleader were constructed to serve as templates for *in vitro* or *in vivo* transcription of leader, delear, trailer and long leader RNAs. Note that these RNAs possessed a 5′GG extension as compared to measles virus leader or trailer RNAs, as a requirement for T7 transcription initiation. Linearized pcDNA3-myc and pSUPER-shGFP [19] were also used for T7 *in vitro* RNA synthesis. VSV *in vitro* transcription was performed as described elsewhere [48]. Radiolabelling was carried out by either performing the T7 *in vitro* transcription in the presence of UTP[α^32^P] (Amersham Biotechnology) or incubating 293T cells for 4 h in a serum free medium supplemented with ^32^P labelled orthophosphate to get radiolabelled cellular RNA. RNA was biotinylated by *in vitro* transcription in the presence of 3.5 mM [biotin-16-UTP] (Roche) and 6.5 mM [UTP] (Promega). RNA purifications were performed with Trizol reagent (Invitrogen). Short size VSV RNA transcripts (microRNAs, <200 nucleotides) were purified using RNeasy Minelute (Qiagen) columns. For cellular nuclear and cytoplasmic RNA preparation, a pellet of 5×10^6^ 293T cells, washed once in PBS, was lysed for 15 min on ice in a buffer containing 25 mM Tris/HCl pH 7.8, 150 mM NaCl, 5 mM EGTA, 1 tablet of Complete (Roche) protease inhibitor per 50 ml, 5 mM Na_3_VO_4_ and 0.5% NP40. Sample was centrifuged for 10 min at 14,000 rpm and the pellet containing the nuclei were washed once with the lysis buffer. RNAs were purified from the nuclei with RNeasy minispin columns with DNase treatment (Qiagen). RNAs were visualised after size separation in 12% acrylamide denaturating gel containing 8 M of urea and ethidium bromide staining. RNase A treatments were done in low salt conditions.

### Transfections

DNA was transfected into 293T cells with Profection Mammalian Transfection System-Calcium Phosphate (Promega). Purified RNAs (200 ng) were transfected with Oligofectamine (Invitrogen). The concentration of the microRNAs (size <200 nucleotides, which is the cut off of RNeasy mini-columns from Qiagen) transcribed *in vitro* from VSV was too low to be quantified using standard procedures and a fixed volume of the preparation was transfected. RNA transfections in Huh7/7.5 cells, were performed with Fugene 6 reagent (Roche). For positive controls, 0.5 µg of poly(I:C) (Amersham) were transfected.

### RNA extraction, cDNA reverse transcription (RT), and real-time QPCR analysis

The procedures and primers sequences have been described in full detail elsewhere [10]. N mRNA was quantified by using a specific set of primers while genome/antigenome was quantified by using primers targeting a L-Tr sequence, which straddles the end of the 3′ most distal L gene on the genome and the genome trailer 5′ end (see Figure S1 for details). Primers used for the quantification of IFN-β transcripts were forward 5′-TGGGAGGATTCTGCATTACC-3′, reverse 5′-CAGCATCTGCTGGTTGAAGA-3′. Results were normalized according to the amounts of 18S rRNA.

### Preparation of 5′-monophosphate leader

A long-leader, containing a 5′ extension of 33 extra nt upstream of measles virus leader sequence, was synthesized *in vitro* by T7 polymerase as described above. 1 volume of Stratascript Reverse Transcriptase (Stratagene) buffer was added together with 0.5 nmol of a DNA oligonucleotide complementary to the 5′extension upstream of the leader sequence, incubated for 5 min at 70°C and annealed for 5 min at RT. The DNA-RNA hybrid was treated with 2 µl of RNase H (Promega) for 1 h at 37°C. Then, 1 µl of RQ1 DNase was added for further 15 min at 37°C before RNA extraction with RNeasy Minelute (Qiagen) columns. RNAs were treated with Calf Intestine Phosphatase (Promega) according to the manufacturer's instructions.

### RIG-I immunoprecipitation and RNA binding

293T cells (8×10^5^) were transfected for 30 h with pEF-BOS RIG-I or pDsRed as mock and lysed in 200 µl of NP-40 buffer (50 mM Tris/HCl pH 8, 150 mM NaCl, 2 mM MgCl_2_, 2 mM CaCl_2_, Complete protease inhibitors, 1% NP40). A preclearing step was performed for the different samples for 1 h at 4°C with 20 µl of an irrelevant antibody, 9E10 anti-myc and anti-mouse Ig antibodies bound to protein G–Sepharose beads. 0.5 µl of anti-Flag M2 antibody (Sigma) was added to the lysates overnight. Flag-RIG-I proteins were immunoprecipitated 3 h at 4°C using anti-mouse Ig antibodies bound to protein G–Sepharose beads which were washed in the lysis buffer before the RNA binding step. Since T7 transcripts had comparable sizes, specific activities of the different RNAs were considered identical. RNA inputs were adjusted in order to get the same input radioactivity between the different samples. Radiolabelled RNAs (adjusted to 10^5^ cpm/assay corresponding to 200 ng for leader RNA and 100 ng for the longer shRNA) were incubated with the beads in 200 µl of lysis buffer supplemented with 1 µg of unlabelled cellular RNA from 293T cells and 2 µl of the RNase inhibitor Rnasin (Promega), for 30 min at RT. Beads were washed 3 times with lysis buffer before liquid scintillation counting. ATP-γS (Sigma) was used at a final concentration of 2 mM.

### RIG-I pulldown by biotinylated RNA

293T cells (4×10^6^) cells were transfected according to Lipofectamine 2000 method (Invitrogen) with 10 µg of pEF-BOS FlagRIG-I, pEF-BOS FlagRIG-I[K270A], pEF-BOS[Q299A] or pDsRed. 48 h post-transfection, cells were lysed in 1 ml of 50 mM Hepes-KOH pH7.5, 150 mM K-acetate, 5 mM Mg-acetate, 0.1% digitonin, 0.5 mM PMSF, Complete® protease inhibitor tablet (Roche) 1×and 2 mM DTT, and sonicated (5×10 s). Insoluble material was removed by centrifuging at 20,000 g for 15 min. 16 µg of biotinylated RNA were added to 350 µg of lysate in binding buffer (20 mM Hepes pH 7.9, 2 mM EDTA, 15% glycerol and 0.05% NP-40), 50 mM NaCl, 1000 units/ml RNasin (Promega), 0.2 mg/ml tRNA (Ambion), 0.5 mM PMSF, Complete® protease inhibitor tablet 1×and 2 mM DTT. The mixture was stirred for 2 h at 4°C. 50 µl of Dynabeads® M-270 Streptavidin (Dynal Biotech, Invitrogen) pre-washed twice and pre-equilibrated for 2 h at 4°C with blocking buffer (base buffer with 100 mM NaCl, 100 units/ml RNasin, 0.2 mg/ml tRNA, 0.1 mg/ml glycogen, 10 mM BSA and 2 mM DTT) was added to the binding mixture and incubated for two additional hours. The complex was washed thrice by incubating for 10 min (with agitation) in wash buffer (binding buffer with 100 units/ml RNasin). Proteins were eluted from the beads by incubating the complex in 25 µl of wash buffer containing 500 mM NaCl. When step gradient elution was performed, the complex was sequentially incubated in wash buffer containing 50, 100, 150, 200, 300 and 500 mM NaCl. Proteins were finally analysed by SDS-PAGE. An anti-Flag M2 antibody (Sigma) was used to assess RIG-I binding to RNA.

### Modelling of RIG-I helicase domain

Using a BLAST [49] search of the RIG-I sequence against the pdb [50], we identified a structural homologue, Hef (pdb code 1WP9) [30]. We then searched RIG-I for the Walker A, Ia, Walker B, III, IV, V, VI helicase motifs [35]. Using the MUSCLE program [51], we performed a multiple sequence alignment among RIG-I, Mda-5 (accession number: Q9BYX4), Hef, PcrA (pdb code 1PJR), a DNA helicase from *Bacillus stearothermophilus*, and Rep (pdb code 1UAA), a DNA helicase from *E. coli*. The resulting alignment was then manually adjusted so as to align the conserved helicase motifs. A good correlation was observed between the RIG-I predicted secondary structure elements (as obtained by using Psipred [52] and those observed in the crystal structure of Hef [30] (see Figure 5A), which points out the reliability of the sequence alignment. This refined sequence alignment was used as a template to build a homology-derived 3D model of the RIG-I helicase domain by using the Swiss-model server [53]. Notably, a threading approach using 3D-PSSM [54] led to a model that is nicely superimposable onto the homology-derived one (rmsd of 1.35 Å, data not shown). The two models share the same overall topology, despite minor structural differences within loops (data not shown). The good agreement between the two models points out the reliability of the structural prediction.

We then performed a multiple structural alignment among the RIG-I model, PcrA, Rep, and the NS3 helicase from HCV (pdb code 1A1V) using the MSDFOLD server [55].

## Supporting Information

Figure S1 Schematic view of measles virus RNAs and recognition by RIG-I. Measles virus genome is an encapsidated negative strand RNA composed of (from 3′ to 5′ end): the leader (Le), containing the transcription and replication promoters, 6 genes encoding in the order of the nucleoprotein (N), the phosphoprotein (P), the matrix protein (M), the fusion protein (F), the hemagglutinin (H), the polymerase (L), and finally the trailer (Tr), which hosts the antigenome promoter. It is transcribed sequentially into non capped non poly-adenylated leader (Le) RNA and into 6 capped and polyadenylated mature mRNAs. The genome is replicated into antigenome, which is essentially a replication template. Encapsidation of the genome and antigenome is represented by grey N blocks with levels of grey indicating gene borders. Note that the gene sizes do not match real ones. The presence of the phosphate group in 5′ end is indicated by “p” letters. RIG-I binds to Leader RNA and the recognition of its 5′ppp end activate RIG-I and downstream signalling leading to IFN response.
(4.14 MB TIF)

Figure S2 Predicted structure of leader, delear and myc short RNAs. Predictions were made according to Zuker's program (www.bioinfo.rpi.edu/applications/mfold/old/rna/form1.cgi).
(4.04 MB TIF)

Figure S3 (A) Strategy used to derived 5′-monophosphate leader (le) RNA from longer 5′-triphosphate RNA leader (lle) precursors synthesized in vitro. (B) Pure RNAs were analysed by electrophoresis on polyacrylamide gel and stained with ethidium bromide. Note that the 5′-triphosphate leader migrated slightly ahead of the 5′-monophosphate leader as expected from its more acidic charge.
(4.43 MB TIF)

Figure S4 Inability of cellular RNA to activate the IFN response. Cytoplasmic and nuclear RNAs extracted from uninfected cells were transfected into 293T cells and analysed for their ability to induce IFN-β response.
(0.49 MB TIF)
