# Supplementary figures and images for: Cytosolic 5′-Triphosphate Ended Viral Leader Transcript of Measles Virus as Activator of the RIG I-Mediated Interferon Response
Source: PLoS One. 2007 Mar 14;2(3):e279. doi: 10.1371/journal.pone.0000279 (PMC1804102; doi:10.1371/journal.pone.0000279)

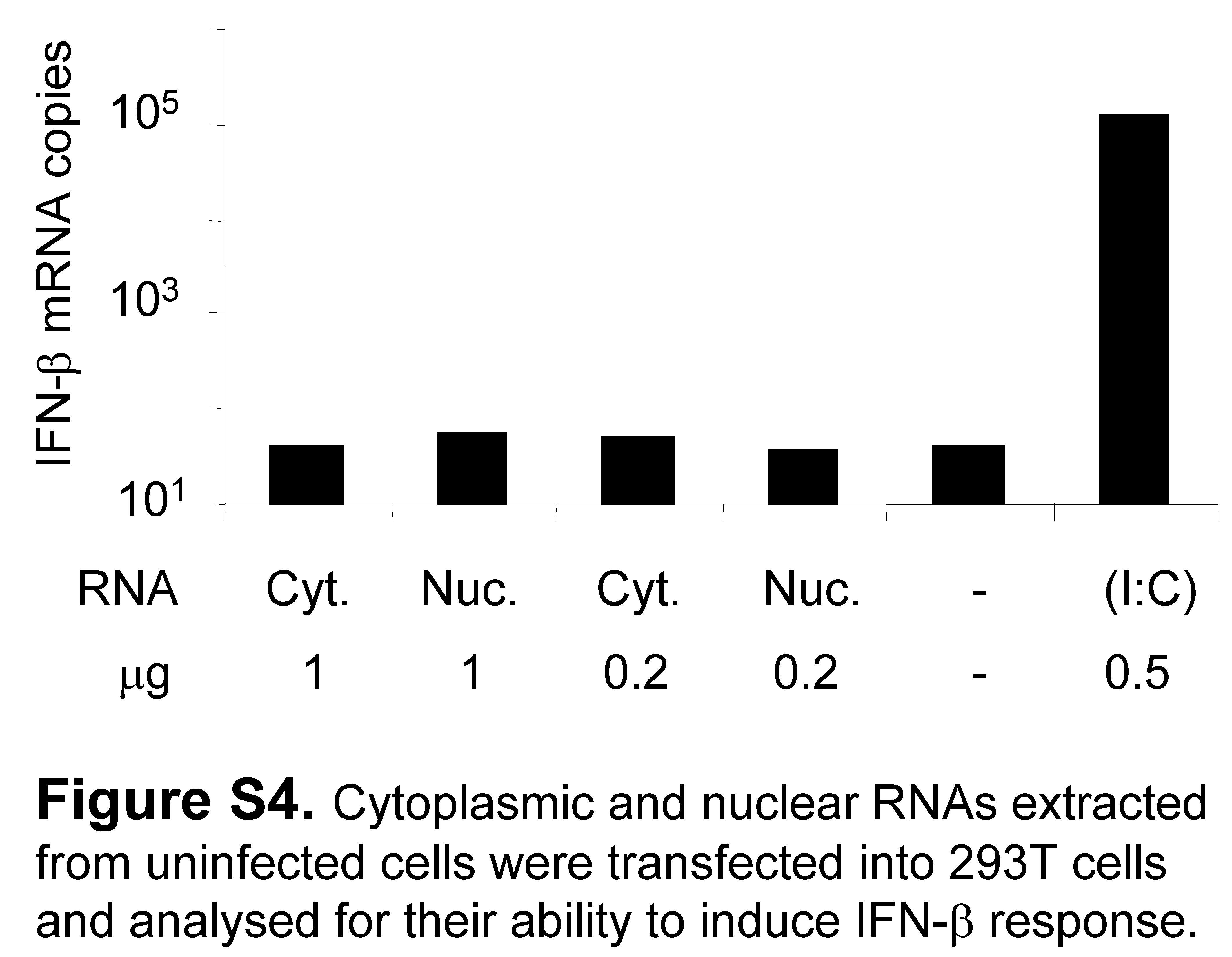

Supplement: Figure S4 — Inability of cellular RNA to activate the IFN response. Cytoplasmic and nuclear RNAs extracted from uninfected cells were transfected into 293T cells and analysed for their ability to induce IFN-β response. (0.49 MB TIF) [file pone.0000279.s004.tif]
